# Thrombospondin-1 mitigates osteoarthritis progression by inhibiting mechanical stress-induced chondrocyte ferroptosis via the integrin/YAP pathway

**DOI:** 10.3389/fimmu.2025.1577234

**Published:** 2025-05-22

**Authors:** Shaoyi Wang, Xiaocong Zhou, Fujian Zhang, Haoxin Zhai, Yuanqiang Zhang, Yongyuan Guo

**Affiliations:** ^1^ Department of Orthopedics, Qilu Hospital, Cheeloo College of Medicine, Shandong University, Jinan, Shandong, China; ^2^ Qilu Hospital of Shandong University Spine and Spinal Cord Disease Research Center - International Chinese Musculoskeletal Research Society (ICMRS) Collaborating Center for Orthopedic Translational Research, Shandong University, Jinan, Shandong, China; ^3^ Health Management Centre, The First Affiliated Hospital of Shandong First Medical University, Jinan, Shandong, China

**Keywords:** osteoarthritis, ferroptosis, mechanical stress, THBS1, chondrocytes

## Abstract

**Introduction:**

Osteoarthritis in weight-bearing joints significantly impacts the quality of life in middle-aged and elderly individuals. Abnormal mechanical stress can induce chondrocytes ferroptosis, thereby accelerating the progression of osteoarthritis. In this study, we investigated the therapeutic effects of targeting chondrocyte ferroptosis to delay the progression of osteoarthritis and identified a potential therapeutic target.

**Methods:**

Through transcriptomic sequencing analysis, we identified a potential association between thrombospondin-1 (THBS1) and mechanical stress-induced chondrocyte ferroptosis. In this study we used via adeno-associated virus-mediated THBS1 overexpression, cell pressurization model and GPX4-conditional knockout (Col2a1-CreERT: GPX4^flox/flox^) mice to verify the regulatory effect of THBS1 on chondrocytes ferroptosis. Additionally, protein interaction network analysis, immunofluorescence co-localization, and co-immunoprecipitation were conducted to investigate the mechanism by which THBS1 modulates chondrocytes ferroptosis.

**Results:**

The expression of THBS1 protein was reduced in load-bearing cartilage tissue in humans. THBS1 suppressed chondrocytes ferroptosis induced by excessive mechanical stress. Immunofluorescence co-localization and CO-IP experiments indicated that integrin αV/β1 serves as the membrane receptor through which THBS1 regulates chondrocyte ferroptosis under mechanical stress. Upon activation, integrin αV/β1 modulated YAP1 nuclear translocation, thereby affecting GPX4 activity. Intra-articular injection of THBS1 synthetic peptides effectively reduced cartilage damage in mouse OA models, protecting articular cartilage and slowing the progression of osteoarthritis.

**Discussion:**

Our results indicate THBS1 regulates mechanical stress-induced chondrocyte ferroptosis through the Integrin/YAP pathway. Furthermore, THBS1 effectively slows the progression of osteoarthritis and protects articular cartilage.

## Introduction

Osteoarthritis (OA) is a kind of degenerative disease involving all joints of the body. With the acceleration of the aging process of the population, the number of patients with osteoarthritis is also increasing year by year, and it has become a common clinical disease (1). At present, drugs can only relieve the painful symptoms of arthritis, but cannot fundamentally block the loss and degeneration of joint cartilage. Therefore, it is necessary to further explore the pathogenesis of osteoarthritis and find effective therapeutic targets in order to effectively block the progress of osteoarthritis.

Abnormal mechanical stress is an important factor to induce osteoarthritis. The exposure of chondrocytes to abnormal mechanical stress causes a series of changes, including apoptosis, necrosis, and ferroptosis. Ferroptosis is a new type of cell death which is characterized by oxidative damage to phospholipids (2). GPX4(Glutathione peroxidase 4) plays a central role in the regulation of ferroptosis, which works with GSH (glutathione) to remove excess ROS in cells and protect the phospholipid structure from oxidative damage (3). The authors’ previous studies found that abnormal mechanical stress induced calcium influx to induce chondrocyte ferroptosis by activating Piezo1 protein (4). In the process of mechanical stress-induced ferroptosis of chondrocytes, we found that THBS1(thrombospondin-1) may be related to this process.

THBS1 is an extracellular matrix protein that plays an important role in cell-matrix interactions (5), which are also important pathways of mechanical signal transduction. THBS1 is considered to be an important ligand in Integrin systems, interacting with various Integrin isoforms such as αVβ1, α3β1, and α5β1 (6, 7). Integrin system is a kind of transmembrane receptor protein, which can mediate adhesion and signal transduction (including mechanical signal transduction) between cells and extracellular matrix, and plays an important role in biological processes such as cell migration, proliferation and differentiation (8). Mechanical stress is an important factor regulating the combination of THBS1 and Integrin. Under mechanical stress, the THBS1 protein in the extracellular matrix was stretched and twisted, causing its conformation to change, exposing integrin-binding sites (9). This structural change enhanced the affinity between THBS1 and Integrin, facilitating the binding between them. THBS1 is expressed in cartilage tissue and its expression is decreased in the tissues of patients with advanced osteoarthritis (10). In addition, studies have shown that the expression of THBS1 in loading area of OA patients is significantly reduced (11). Some researchers have also found a potential role for THBS1 in the treatment of osteoarthritis (12).

In this study we plan to analyze the role of THBS1 in the progression of osteoarthritis. To explore the relationship between THBS1 and chondrocyte ferroptosis induced by mechanical stress. To elucidate the mechanism of THBS1 regulating chondrocyte ferroptosis. In addition, we will also determine the therapeutic effect of THBS1 on osteoarthritis.

## Materials and methods

### Animals

All procedures involving animals in this research were carried out under the approval and guidance of the Laboratory Animal Centre at Qilu Hospital, Shandong University (Approval No. KYLL-2022(ZM)-136). The Col2a1-CreERT transgenic mice, developed by Cyagen (USA), were acquired from the same institution. GPX4^flox/+^ mice were also engineered by Cyagen using ES cell technology. To establish the experimental model, GPX4^flox/+^ mice were bred to yield homozygous GPX4^flox/flox^ offspring. These homozygous GPX4^flox/flox^ mice were then crossed with Col2a1-CreERT carriers, producing progeny with both the Col2a1-CreERT and GPX4^flox/+^ genotypes. By mating Col2a1-CreERT GPX4^flox/+^ mice, we obtained Col2a1-CreERT GPX4^flox/flox^ mice (**Supplementary Figure S2A**). Only male mice with the Col2a1-CreERT and GPX4^flox/flox^ genotypes were used for experimental studies, while Col2a1-CreERT ^GPX4+/+^ littermates were designated as the wild-type (WT) control group (13). Ten-week-old Col2a1-CreERT GPX4^flox/flox^ mice were intraperitoneally injected with tamoxifen (1 mg/d*5 d) (MCE, USA, Cat#HY-13757A) to obtain GPX4-conditional knockout (GPX4-CKO) mice (13).

### Genotyping

Tail samples were collected from 4-week-old mice, and genomic DNA was extracted using the One Step Mouse Genotyping Kit (Vazyme, China, Cat# PD101-01) following the manufacturer’s guidelines. Primers specific to GPX4^flox^ and Col2a1-CreERT sequences, detailed in **Supplementary Table S1**, were used for PCR amplification. For gel preparation, 1.5 g of agarose was combined with 100 mL of 2× Tris-acetate-EDTA (TAE) buffer and 6 µl of Gel Red, then heated until fully dissolved. The PCR products were separated by agarose gel electrophoresis, and bands were visualized with an Amersham Imager 680 (GE, USA). For the Col2a1-CreERT positive genotype, a 358 bp band was observed, while no band indicated a wild-type genotype. GPX4^flox/flox^ was identified by a 238 bp band, GPX4^+/+^ by a 204 bp band, and GPX4^flox/+^ by bands at both 238 and 204 bp. Refer to **Supplementary Table S1** for primers and **Supplementary Figures S2B, C** for genotyping results.

### Animals

All animal procedures in this study were conducted following institutional regulations and received approval from the Laboratory Animal Center of Qilu Hospital, Shandong University (Approval No. KYLL-2022(ZM)-136). The C57/BL6 wild-type (WT) mice were obtained from the Experimental Animal Center of Shandong University, while the Wistar rats were sourced from Vital River Laboratory Animal Technology, Beijing, China.

Eight-week-old Wistar rats were randomly assigned to three groups: CON, AAV-GFP, and AAV-THBS1, with each group consisting of 10 animals. All rats were anesthetized using pentobarbital, and a 5 mm incision was made on the lateral side of the knee joint to expose the joint. The AAV-THBS1 group received an injection of 50 µl of AAV-THBS1 (1.5×10^12 vg/ml, GENE, China) into the medial and lateral spaces anterior to the knee joint. The AAV-GFP group was injected with the same volume and concentration of AAV-GFP at the same site, while the CON group was administered an equivalent volume of PBS. After the procedure, the incision was sutured and disinfected daily until healing was complete. Fourteen days post-surgery, *in vivo* 3D optical imaging was performed using the IVIS Spectrum system (PerkinElmer, USA) to assess transfection efficiency. From each group, three rats with successful transfection were selected, and knee joint tissues were collected and processed for tissue sectioning. The expression of THBS1 was then evaluated through immunofluorescence staining and Real-time PCR.

To verify the therapeutic effect of THBS1 on osteoarthritis, we selected ABT-510, a THBS1-like peptide. Twelve-week-old mice were divided into three groups: CON group, PBS group, and ABT-510 group, with 10 mice in each group. The mice in the PBS and ABT-510 groups underwent the DMM model in the left knee, and 10 µL of PBS or ABT-510 (14) (60 mg/kg) was injected biweekly for a total of four injections.

Knee joints were collected from all animals eight weeks after the DMM model was established. Mice exhibiting infections, tumors, or poor overall health were excluded from the analysis. To account for potential losses due to infection, an additional three to four mice were included in each group as a precaution.

The experiment was designed with randomization in mind, with male mice of comparable age and weight randomly assigned to different groups. After assignment, the number of mice in each group was either equal or showed no statistically significant difference, thereby minimizing experimental bias.

For each experiment, the sample size included a minimum of three mice per condition. Data are expressed as mean ± standard deviation. Group comparisons were conducted using one-way analysis of variance (ANOVA) through GraphPad Prism 7 (GraphPad Software Inc., San Diego, CA, USA).

Animals are euthanized using pentobarbital anesthesia. Animals were anesthetized with pentobarbital and killed for cervical dislocation. Pentobarbital was provided by Animal Experimental Center of Shandong University.

### Surgically induced OA model

Mice and rats were anesthetized using pentobarbital, and the destabilization of the medial meniscus (DMM) surgery was carried out under a microscope for precision. Following the procedure, the incisions were sutured and disinfected daily until full recovery. All animals were kept in standard housing conditions, with precautions taken to prevent any biting or interference with the incision sites.

### *In vitro* mechanical stress culture model

We designed the *in vitro* cell pressurization device based on relevant literature (15, 16). For accompanying images and detailed operating procedures, please refer to our previously published articles (4). The procedure is described as follows:

The cells were pre-seeded onto 14- or 24-mm cell slides, which were then positioned on the scaffold. Both the cell slides and scaffolds were placed in a sealed chamber filled with complete culture medium. To pressurize the liquid inside the chamber, a flexible rubber sheet was applied. Chondrocytes were subjected to mechanical stress at 1 MPa and a frequency of 1 Hz for 1 hour, using a pneumatic device (FESTO, Germany), as the average pressure on mouse knee cartilage is approximately 0.4 MPa. Prior to the experiment, the chamber containing the culture medium was placed in a cell incubator for 6 hours to ensure cell viability. After the pressure was applied, the cells or femoral heads were transferred to fresh complete medium for further analysis.

### Primary cell isolation and culture

Primary chondrocytes from mice were isolated as previously described (17, 18). In brief, cartilage from the distal femur and proximal tibia of 5-day-old mice was carefully harvested under a microscope. The cartilage was then digested with 0.2% collagenase type II (Gibco) at 37°C for 8 hours. The resulting chondrocytes were plated at a density of 5.7×10^5 cells/cm² and cultured in DMEM/F12 (HyClone, Logan, USA) supplemented with 10% fetal bovine serum (FBS; Gibco, USA), 100 U/ml penicillin, and 0.1 mg/ml streptomycin (HyClone, USA). The cells were incubated under standard conditions (37°C, 5% CO2), and the culture medium was refreshed every 3 days. Chondrocytes from up to the fifth passage were used for all *in vitro* experiments, with two replicate wells used for each experiment.

### Drugs and cell culture reagents

Recombinant human Thrombospondin-1 protein (rhTHBS1) was obtained from MCE (HY-P70725). RhTHBS1(100ng/ml) (19) was added to human chondrocytes at the same time of mechanical stress stimulation *in vitro*, and incubated for 24 hours after the end of mechanical stress stimulation. IntegrinαVβ1 inhibitor αVβ1 integrin-IN- 1 was obtained from MCE (HY-145363). αVβ1 integrin-IN- 1(100ng/ml) (20) was added to chondrocytes at the same time of mechanical stress stimulation with or without rhTHBS1, and incubated for 24 hours after the end of mechanical stress stimulation. YAP1 inhibitor PROTAC YAP d1 was obtained from MCE (HY-168016). PROTAC YAP d1(20μM) (21) was added to chondrocytes at the same time of mechanical stress stimulation with or without rhTHBS1, and incubated for 24 hours after the end of mechanical stress stimulation.

### Knockdown of THBS1 by siRNA

To knock down THBS1, chondrocytes were transfected with 100nM siTHBS1 and scrambled control siRNA (scRNAi) by using the siRNA transfection reagent PROTPCOL (Polyplus, France) (22). After 3 days, Transfection efficiency was assessed by WB. After incubation with siTHBS1 or scRNAi for 3 days, chondrocytes were treated. The sequences of siRNA and Realtime-PCR are shown in **Table 1**.

**Table 1:** Primers used for si-THBS1.

Target	Forward Primers,5’-3’	Reverse Primers,5’-3’
scRNAi	UUCUCCGAACGUGUCACGUTT	ACGUGACACGUUCGGAGAATT
siTHBS1-1	GCGUGAAGUGUACUAGCUATT	UAGCUAGUACACUUCACGCTT
siTHBS1-2	CCAACAAACAGGUGUGCAATT	UUGCACACCUGUUUGUUGGTT

### Immunohistochemistry

Eight weeks after the DMM model was established, the knee joints were harvested. The tissue samples were fixed in 4% paraformaldehyde, decalcified using 10% ethylenediaminetetraacetic acid (EDTA), and sectioned into 5µm slices. Human cartilage was directly prepared into 5µm sections after paraffin embedding. Paraffin-embedded sections were deparaffinized with xylene and gradient ethanol. Antigen retrieval was performed using citric acid (pH 6.0), followed by blocking with Bovine Serum Albumin (BSA). The sections were incubated overnight at 4°C with the following primary antibodies: rabbit anti-THBS1 (1:100, ab267388, Abcam, USA), rabbit anti-Aggrecan (1:1000, 13880-1-AP, Proteintech, USA), rabbit anti-Col2 (1:1000, 28459-1-AP, Proteintech, USA), rabbit anti-ADAMTS-5 (1:1000, ab231595, Abcam, USA), rabbit anti-MMP-9 (1:1000, ab283575, Abcam, USA). The next day, the sections were incubated with a secondary goat anti-rabbit IgG-HRP antibody (1:200, 111-005-003, Jackson ImmunoResearch, USA) for 60 minutes at room temperature. Tissue staining was visualized under an IX71-SIF microscope (Olympus, Tokyo, Japan), with brown particles indicating positive staining. Image analysis was conducted using Image-Pro Plus 6.0 software (Media Cybernetics, Inc., USA).

Additionally, Safranin O/Fast Green staining was carried out using a modified Safranin O/Fast Green FCF cartilage staining kit (Solarbio, Beijing, China), following the manufacturer’s protocol.

Hematoxylin-Eosin (H&E) staining, after being deparaffinized and rehydrated as usual, sections were stained with Hematoxylin-Eosin/HE Staining Kit (Solarbio, Beijing, China) according to the instructions.

### Histopathologic and quantificational evaluation of OA

The Osteoarthritis Research Society International (OARSI) histological scoring system was employed to assess the proteoglycan content in articular cartilage, based on Safranin O staining (23) (**Supplementary Table S2**).

### Immunofluorescence staining

The cells were treated as specified, and either fixed immediately or after a 24-hour period with 4% paraformaldehyde. They were then permeabilized with 0.2% Triton-X 100 for 20 minutes and blocked with 1% BSA for 30 minutes. Following this, the cells were incubated overnight at 4°C with various primary antibodies: rabbit anti-YAP1 (1:200, 13584-1-1AP, Proteintech, USA), rabbit anti-IntegrinαV (1:200, ab150361, Abcam, USA), rabbit anti-Integrinβ1 (1:200, ab183666, Abcam, USA), rabbit anti-Integrinβ3 (1:200, ab179473, Abcam, USA), rabbit anti-CD36 (1:200, ab252922, Abcam, USA), rabbit anti-CD47 (1:200, ab319049, Abcam, USA), and rabbit anti-THBS1 (1:200, ab267388, Abcam, USA). On the following day, the cells were treated with fluorescently labeled goat anti-rabbit IgG (1:100, A23220 and A23320, Abbkine, China) for 1 hour. Images were captured using an IX71-SIF microscope (Olympus, Tokyo, Japan) and analyzed with Image-Pro Plus 6.0 software (Media Cybernetics, Inc., USA).

The immunofluorescence protocol for tissue sections closely followed that of immunohistochemistry. Specifically, sections were incubated overnight at 4°C with rabbit anti-THBS1 (1:100, ab267388, Abcam, USA) on the first day. On the second day, sections were exposed to fluorescently labeled goat anti-rabbit IgG (1:100, A23320, Abbkine, China) for 1 hour. Images were acquired using an IX71-SIF microscope (Olympus, Tokyo, Japan) and analyzed with Image-Pro Plus 6.0 software (Media Cybernetics, Inc., USA).

### Co-immunoprecipitation

Total protein was extracted from chondrocytes immediately following stimulation with high mechanical stress *in vitro*. The cells were placed in RIPA lysis buffer (Millipore, Billerica, MA, USA) containing 5% PMSF (a protease inhibitor) and incubated on ice for 40 minutes. After centrifugation at 12,000 rpm for 15 minutes at 4°C, the supernatant was collected. To eliminate non-specific protein binding to the beads, 4 µL of Protein A/G PLUS-agarose (Santa Cruz, USA) and 200 µL of pre-cleared lysates were incubated with 0.4 µL of immunoglobulins (IgGs) from the same species as the primary antibody (rabbit anti-goat IgGs, ZB2306, Zsbio, China) for 1 hour at 4°C. The total protein lysates were then centrifuged at 12,000 rpm for 15 minutes at 4°C, and the supernatant was retained. The protein concentration was quantified using a BCA protein assay kit (Biotechnology Co, Beijing, China) following the manufacturer’s instructions, and the total protein concentration was adjusted to 1 µg/µL. Subsequently, 200 µL of total protein lysates and 8 µL of Protein A/G PLUS-agarose (Santa Cruz, USA) were incubated with 2 µL of primary antibody (rabbit anti-THBS1, 1:200, ab267388, Abcam, USA) on ice with shaking overnight. The following day, the beads were washed three times with pre-cooled PBS. The bound proteins were then mixed with 50 µL of SDS-PAGE Sample Loading Buffer (Beyotime, China) and boiled at 95°C for 10 minutes before proceeding with Western blot analysis.

### Total protein extraction and Western blotting

Chondrocytes were treated according to the specified protocol. After either immediate treatment or a 24-hour incubation, the cells were lysed in RIPA lysis buffer (Millipore, Billerica, USA) containing 1 mM PMSF (Beyotime, China) and kept on ice for 30 minutes. The lysate was then centrifuged at 12,000 rpm for 15 minutes at 4°C, and the supernatant was collected. To ensure equal protein amounts across groups, a BCA protein assay kit (Biotechnology Co, China) was utilized. Proteins from each group were separated using 10% SDS–polyacrylamide gel electrophoresis (SDS-PAGE) and subsequently transferred to a polyvinylidene difluoride (PVDF) membrane (Millipore, USA). The PVDF membranes were blocked in Tris-buffered saline with Tween-20 (TBST) containing 5% milk powder for 1 hour. They were then incubated overnight at 4°C with the following primary antibodies: rabbit anti-GPX4 (1:1000, ab125066, Abcam, USA), rabbit anti-Aggrecan (1:1000, 13880-1-AP, Proteintech, USA), rabbit anti-THBS1(1:500, A2125, ABclonal, China), rabbit anti-Col2 (1:1000, 28459-1-AP, Proteintech, USA), rabbit anti-ADAMTS-5 (1:1000, ab231595, Abcam, USA), rabbit anti-MMP-9 (1:1000, ab283575, Abcam, USA), and rabbit anti-GAPDH-HRP (1:5000, 10494-1-1AP, Proteintech, USA). On the following day, the membranes were washed with TBST and then incubated with goat anti-rabbit IgG-HRP secondary antibody (1:5000, 111-005-003, Jackson ImmunoResearch, USA) for 1 hour at room temperature. Bands were visualized using a FluorChem E Chemiluminescent Western Blot Imaging System (Amersham Imager 600, USA) and quantified using Image-Pro Plus 6.0 software (Media Cybernetics, Inc., USA).

### RNA extraction and real-time PCR

Femoral head tissues, including mouse cartilage, were processed as specified. After a 24-hour period, the tissues were ground into a powder using liquid nitrogen. Similarly, chondrocytes were treated as indicated, and they were collected after 24 hours. For RNA extraction from both the cells and the powdered tissues, TRIzol reagent (Takara Bio, Japan) was utilized. A total of 1 µg of RNA was reverse-transcribed into complementary DNA (cDNA) using a cDNA Synthesis Kit (Toyobo, Japan). Real-time PCR was conducted using SYBR Green PCR Matrix Mix (Toyobo, Japan) on a Bio-Rad thermal cycler (Hercules, USA) under the following cycling conditions: an initial denaturation at 95°C for 1 minute, followed by 40 cycles of 95°C for 15 seconds, 60°C for 15 seconds, and 72°C for 45 seconds, ending with an extension at 72°C for 5 minutes. The RT-PCR results were calculated using the 2-ΔΔCt method. The specific primers used in the experiment are listed in **Table 2**.

**Table 2:** Real-Time PCR primers.

Target	Forward Primers,5’-3’	Reverse Primers,5’-3’
THBS1	GAAGCAACAAGTGGTGTCAGT	ACAGTCTATGTAGAGTTGAGCCC
Col2a1	CCAGATTGAGAGCATCCGCA	ACTTTCATGGCGTCCAAGGT
GPX4	GTGTAAATGGGGACGATGCC	ACCACGCAGCCGTTCTTATC
β-actin	TCACCCACA CTGTGCCTATCTACGA	CAGCGGAACCGCTCATTGCCAATGG
ADAMTS-5	ACTTTGTTGCCAATTCCAGG	TTTGAGAACACGGGGAAGAC
Aggrecan	AAACCTGGCGTGAGAACTGT	CCACTGACACACCTCGGAAG
MMP-9	TTGACAGCGACAAGAAGTGG	GCCATTCACGTCGTCCTTAT

### Construction of protein interaction network

The interaction between THBS1 and other protein was analyzed by the application of the STRING database (https://string‐db.org/).

### Human samples and ethics statement

Human knee cartilage samples were obtained from six male patients who underwent unilateral total knee arthroplasty due to osteoarthritis at Qilu Hospital of Shandong University. The patients included: Patient 1 (57 years old, BMI=24.8), Patient 2 (60 years old, BMI=26.3), Patient 3 (62 years old, BMI=25.1), Patient 4 (60 years old, BMI=23.9), Patient 5 (62 years old, BMI=25.1), and Patient 6 (59 years old, BMI=24.9). All patients were free of other systemic diseases. The knee joint tissues from each patient were categorized into two groups: the loading group and the non-loading group. A portion of the tissue was stored in an electron microscope fixing solution (Servicebio, Cat# G1102) for transmission electron microscopy (TEM) and another portion was stored in 4% polyformaldehyde for paraffin section preparation. All participants provided informed consent, and the study received ethical approval from the Medical Ethical Committee of Qilu Hospital of Shandong University (KYLL-2022(ZM)-136).

### Microarray RNA-sequence

Human knee cartilage samples were stored in RNA Keeper and transported on dry ice for analysis. Comprehensive gene expression profiling was conducted by Qinglian Biotech (Beijing, China). To explore the connections between mechanical stress and osteoarthritis, Kyoto Encyclopedia of Genes and Genomes (KEGG) pathway analysis and Gene Ontology (GO) term enrichment were utilized.

### Transmission electron microscopy

Human knee cartilage tissues were collected into the loading group and the non-loading group described previously. Mouse chondrocytes were stimulated according to the specified protocol. After a 24-hour period, cells were collected using a cell brush, resulting in a total cell count exceeding 6 × 10^5. For preservation and transport, TEM fixative was applied at 4°C. Both chondrocytes and human knee cartilage samples underwent centrifugation and were washed three times with 0.1 M phosphate buffer (pH 7.4). A 1% agarose solution was prepared in advance by heating and dissolving the agarose. The samples, along with human cartilage tissues, were then post-fixed in 1% osmium tetroxide (OsO4) within 0.1 M phosphate buffer (pH 7.4) for 2 hours at room temperature. Following post-fixation, the samples were dehydrated at room temperature using a graded series of ethanol solutions. They were subsequently infiltrated and embedded in Embed 812 resin (SPI, Cat# 90529-77-4) overnight at 37°C. The embedding molds containing the resin and samples were transferred to 65°C ovens for polymerization, which lasted over 48 hours. Resin blocks were then sectioned to a thickness of 60–80 nm using an ultramicrotome (Leica, Cat# Leica UC7) and stained with a saturated alcohol solution of 2% uranyl acetate and 2.6% lead citrate. Finally, the cells were examined using a transmission electron microscope (HT7700, Hitachi, Tokyo, Japan), and images were captured.

### Micro-CT

The scanning protocol utilized an isotropic resolution of 15 μm, with X-ray energy parameters set at 70 kV and 200 μA. The microstructural analysis of the vertebrae was conducted using a Quantum GX2 scanner (PerkinElmer, USA). Before undergoing histological processing, samples were fixed in paraformaldehyde for subsequent micro-CT imaging. The scanned images from each group were assessed using a consistent threshold to facilitate the three-dimensional reconstruction of the structural characteristics of each sample.

### Cell live/dead assay

Cell viability assays were conducted using a Calcein/PI Cell Viability/Cytotoxicity Assay Kit (Beyotime, China, Cat# C2015). Following the indicated treatments, cells were incubated for 24 hours and then stained with calcein AM/PI working solution at 37°C for 30 minutes. Observations were made using an LSM780 laser scanning confocal microscope (ZEISS, Germany). Living cells were indicated by green fluorescence from calcein, while propidium iodide (PI) emitted red fluorescence to label dead cells. Image analysis involved calculating the ratio of dead cells to live cells in three randomly selected fields (24).

### Total reactive oxygen species measurement

Following the specified treatments, cells were incubated with 10 μM 2′,7′-dichlorodihydrofluorescein diacetate (DCFDA, Beyotime, Cat# S0033S) for 30 minutes. After incubation, excess DCFDA was washed away by rinsing the cells twice with PBS. The labeled cells were then trypsinized and resuspended in PBS containing 5% FBS. The conversion of DCFDA to the highly fluorescent compound 2′,7′-dichlorofluorescein (DCF) serves as an indicator of reactive oxygen species (ROS) generation and was analyzed using an LSM780 laser scanning confocal microscope (ZEISS, Germany). High levels of ROS were indicated by increased green fluorescence.

### Analysis of GSH level

Mouse chondrocytes were subjected to the specified stimulation protocols. After 24 hours, cell lysates from the designated groups were obtained to assess glutathione (GSH) levels utilizing a Total Glutathione Peroxidase Assay Kit (Cat# S0058; Beyotime Biotechnology), following the manufacturer’s guidelines as previously described. Data for the analysis were acquired using a Varioskan Flash multifunction plate reader (Thermo Fisher Scientific, Waltham, MA). The concentrations of cytokines (µM) were determined by referencing a standard curve generated with CELLQUEST software.

### JC-1 assay

Mouse chondrocytes were subjected to the specified stimulation conditions. After a 24-hour period, the mitochondrial membrane potential was assessed using a JC-1 assay kit (Beyotime, China, Cat# C2006), in accordance with the manufacturer’s protocol. The concentration of the JC-1 used in the experiment is 10 ug/ml. Following stimulation, the chondrocytes were incubated with a JC-1 staining solution at 37°C for 20 minutes. When the mitochondrial membrane potential is elevated, JC-1 aggregates form, resulting in red fluorescence, whereas lower membrane potential leads to the formation of JC-1 monomers, which emit green fluorescence. Fluorescent images were captured using an LSM780 laser scanning confocal microscope (ZEISS, Germany), and the ratio of red to green fluorescence was used to evaluate alterations in the mitochondrial membrane potential.

### MitoTracker assay

Mouse chondrocytes were treated according to the specified protocol. After 24 hours, MitoTracker Red CMXRos (Beyotime, China, Cat# C1035) was utilized to identify functionally active mitochondria. Following the indicated stimulation, the chondrocytes were incubated with the MitoTracker Red CMXRos solution at 37°C for 30 minutes. Fluorescent images were captured using an LSM780 laser scanning confocal microscope (ZEISS, Germany). The level of red fluorescence intensity was indicative of mitochondrial activity.

### Ethics statement

All animal procedures in this study were conducted following institutional regulations and received approval from the Laboratory Animal Center of Qilu Hospital, Shandong University (Approval No. KYLL-2022(ZM)-136, Title of the approved project: Thrombospondin-1 Mitigates Osteoarthritis Progression by Inhibiting Mechanical stress-induced Chondrocyte Ferroptosis via the Integrin/YAP Pathway. Date of approval: 2022.01.01). And the work has been reported in line with the ARRIVE guidelines 2.0.

All human participants provided informed consent, and the study received ethical approval from the Medical Ethical Committee of Qilu Hospital of Shandong University (Approval No. KYLL-2022(ZM)-136, Title of the approved project: Thrombospondin-1 Mitigates Osteoarthritis Progression by Inhibiting Mechanical stress-induced Chondrocyte Ferroptosis via the Integrin/YAP Pathway. Date of approval: 2022.01.01).

### Statistical analyses

Statistical analyses of all data were conducted using GraphPad Prism 7 (GraphPad Software Inc., San Diego, CA, USA). For comparisons among multiple groups, either t-tests or one-way/two-way ANOVA were employed. The results are presented as mean values ± standard deviation (SD), with a significance threshold set at P < 0.05. Each cell experiment was conducted a minimum of three times to ensure reliability.

## Results

### The expression of THBS1 is diminished in loading regions of the cartilage in patients with knee osteoarthritis

**Figure 1:**
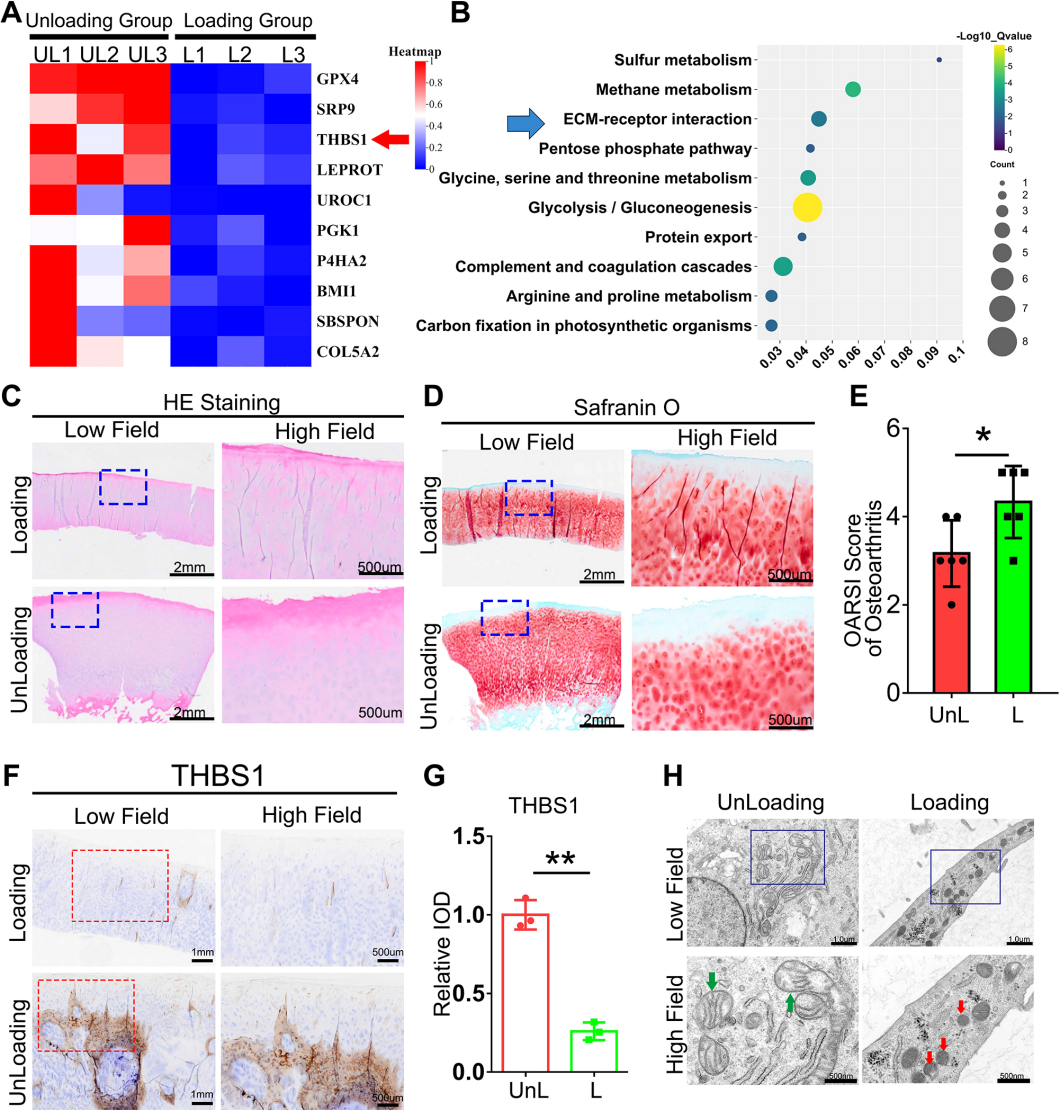
The expression of THBS1 is diminished in loading regions of the cartilage in patients with knee osteoarthritis. **(A)** The Heat map of the TOP10 decreased genes between Loading Group (cartilage tissue samples were obtained from weight-bearing) and Unloading Group (cartilage tissue samples from non-weight-bearing areas). **(B)** The TOP10 GO Enrichment of Loading and Unloading group from human cartilage tissues. **(C)** Representative images of HE staining of cartilage tissues in loading area and unloading area of OA patients. Scale bars, 2 mm (low field), 500 μm (high field). **(D)** Representative images of Safranin O staining of cartilage tissues in loading area and unloading area from OA patients. Scale bars, 2 mm (low field), 500 μm (high field). **(E)** Osteoarthritis Research Society International (OARSI) grade, based on safranin O staining (n=6 for each group). **(F)** Immunohistochemical assay of THBS1 in n loading area and unloading area of OA patients. Scale bars, 1 mm (low field), 500 μm (high field). **(G)** Quantification of immunohistochemical analysis (n=3 for each group). **(H)** Representative TEM images of human chondrocytes from loading area and unloading area of OA patients. (n=3 for each group). Green arrows show the normal mitochondria. Red arrows show the shrunken mitochondria. Scale bars, 1 μm (low field), 500 nm (high field). Data were presented as the mean ± SD. *P<0.05, **P<0.01.

Cartilage tissue samples were obtained from both weight-bearing and non-weight-bearing areas of patients with knee osteoarthritis and subsequently subjected to transcriptome sequencing analysis. As shown in **Figure 1A**, THBS1 expression is reduced in the loading area of cartilage tissue in OA patients. Further enrichment analysis revealed significant enrichment in extracellular matrix (ECM) and receptor interactions (**Figure 1B**). THBS1 has been reported to play a pivotal role in cell-ECM interactions (5). These findings suggest a potential association between THBS1 and mechanical stress-induced chondrocyte damage. Therefore, we further analyzed THBS1 expression in cartilage tissue sections from OA patients. We prepared cartilage tissue sections from both the loading and unloading areas of patients with knee osteoarthritis. Hematoxylin and eosin (HE) staining analysis revealed multiple cartilage fissures in the tissue from the loading area (**Figure 1C**). Safranin-fast green (Safranin O) staining analysis indicated severe cartilage damage in the loading area (**Figures 1D, E**). Importantly, immunohistochemical staining analysis demonstrated that the expression of THBS1 was reduced in the loading area (**Figures 1F, G**). Additionally, transmission electron microscopy analyses of the cartilage tissue in loading area revealed that the nuclei of the mitochondria within chondrocytes were shrunken and that the mitochondrial membranes were thickened, indicating signs of ferroptosis (**Figure 1H**).

### THBS1 protects articular cartilage and delays the progression of osteoarthritis

**Figure 2:**
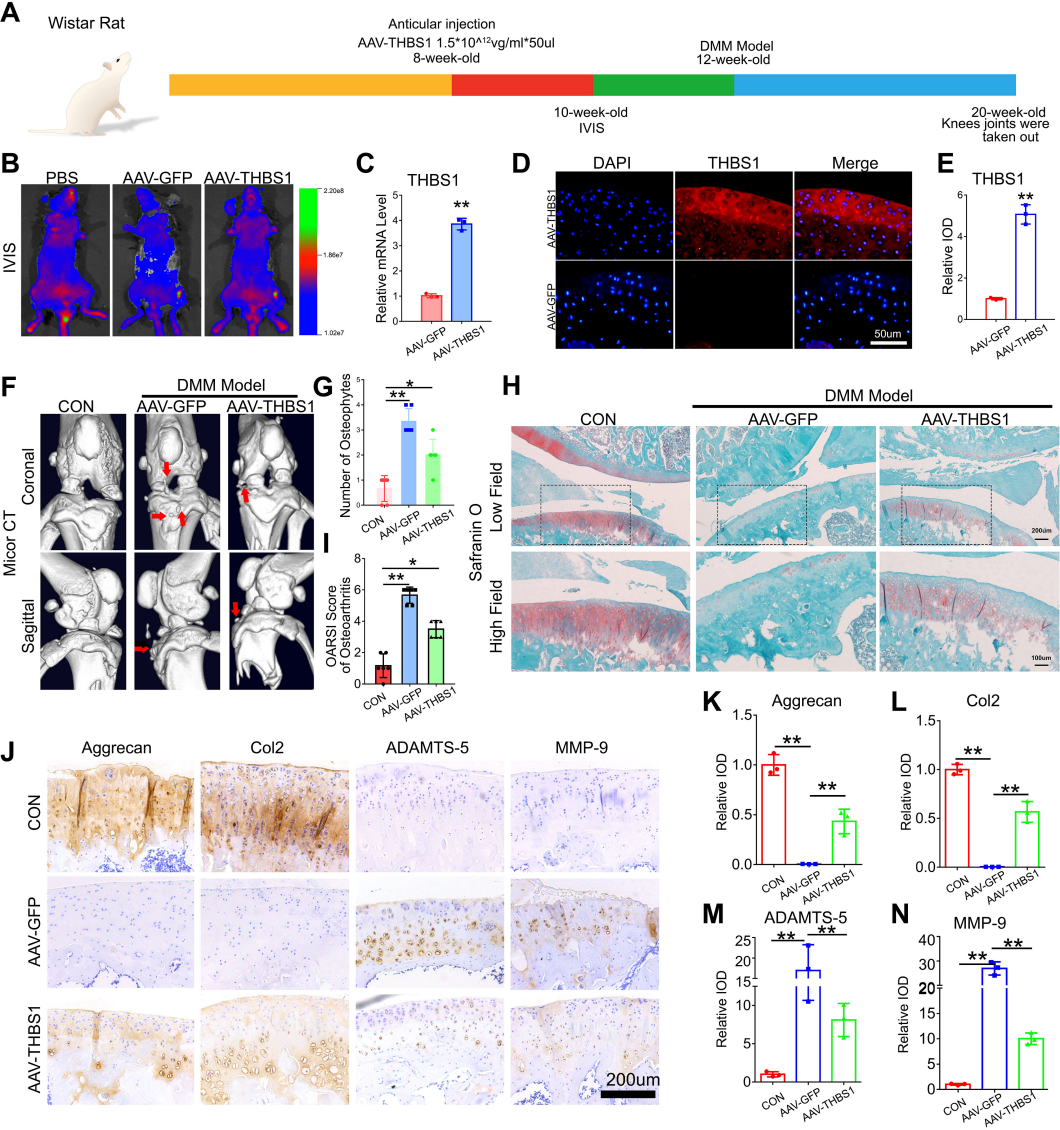
THBS1 protects articular cartilage and delays the progression of osteoarthritis. **(A)** Flowchart of animal experiment. **(B)** *In vivo* imaging system (IVIS) images of 10-week-old ray that were delivered with AAV-GFP, AAV-THBS1 or PBS. **(C)** Real-time PCR of THBS1 in articular cartilage of the AAV-GFP group and AAV-THBS1 group. **(D)** Representative immunofluorescence images of THBS1 in articular cartilage of the indicated groups. Scale bars, 50 μm. **(E)** Quantification of immunofluorescence analysis (n=3 for each group). **(F)** Representative images of Micro-CT of the AAV-GFP group, AAV-THBS1 group or CON group (n=6). Arrows show the formation of osteophytes. **(G)** Osteophyte number assay based on Micro-CT (n=6 for each group). **(H)** Representative images of safranin O staining of the AAV-GFP group, AAV-THBS1 group or CON group. Scale bars, 200 μm (low field), 100 μm (high field). **(I)** Osteoarthritis Research Society International (OARSI) score of OA based on the results of safranin O staining (n=6 for each group). **(J)** Immunohistochemical assay of Aggrecan, Col2, ADAMTS-5 and MMP-9 in articular cartilage of the indicated groups. Scale bars 200 μm. **(K-N)** Quantification of immunohistochemical analysis (n=3 for each group). Data were presented as the mean ± SD. *P<0.05, **P<0.01.

To further investigate the role of THBS1 in the progression of osteoarthritis, we constructed an adeno-associated virus that overexpresses THBS1. Wistar rats were selected and divided into three groups: the CON group, the AAV-GFP group, and the AAV-THBS1 group, with 10 rats in each group. When the rats reached 8 weeks of age, AAV-THBS1 and AAV-GFP were injected into the injection cavity of the left knee joint, while the CON group received an equivalent volume of PBS. The experimental process is illustrated in **Figure 2A**. Two weeks post-injection, IVIS detection was conducted, revealing high signals in the left knee joint cavity for both the AAV-THBS1 and AAV-GFP groups, confirming the success of the injection (**Figure 2B**). Four weeks after injection, three rats were randomly selected from the AAV-THBS1 and AAV-GFP groups, knee joint tissue sections were collected, and the expression of THBS1 was assessed using IF and Real-time PCR methods. As shown in **Figures 2C–E**, the transcription level of THBS1 was elevated in the AAV-THBS1 group, with expression levels also surpassing those in the AAV-GFP group. At 12 weeks of age, the remaining rats in the AAV-GFP and AAV-THBS1 groups underwent DMM model in the left knee. Eight weeks following the establishment of the DMM model, knee joint tissue was extracted for subsequent analyses. Micro CT imaging revealed significant osteophyte proliferation in the knee joint tissue of the AAV-GFP group after osteoarthritis induction via the DMM model (**Figures 2F, G**). In contrast, overexpression of THBS1 effectively inhibited osteophyte proliferation. Safranin O staining indicated severe cartilage loss in the AAV-GFP group; however, overexpression of THBS1 alleviated the extent of cartilage loss, leading to a reduction in the OARSI score (**Figures 2H, I**). IHC analysis demonstrated that THBS1 overexpression resulted in increased expression of anabolic markers, including Col2 and Aggrecan, in cartilage tissue, while the expression of catabolic markers, such as MMP-9 and ADAMTS-5, was decreased (**Figures 2J–N**).

### THBS1 inhibits chondrocytes ferroptosis induced by excessive mechanical stress

**Figure 3:**
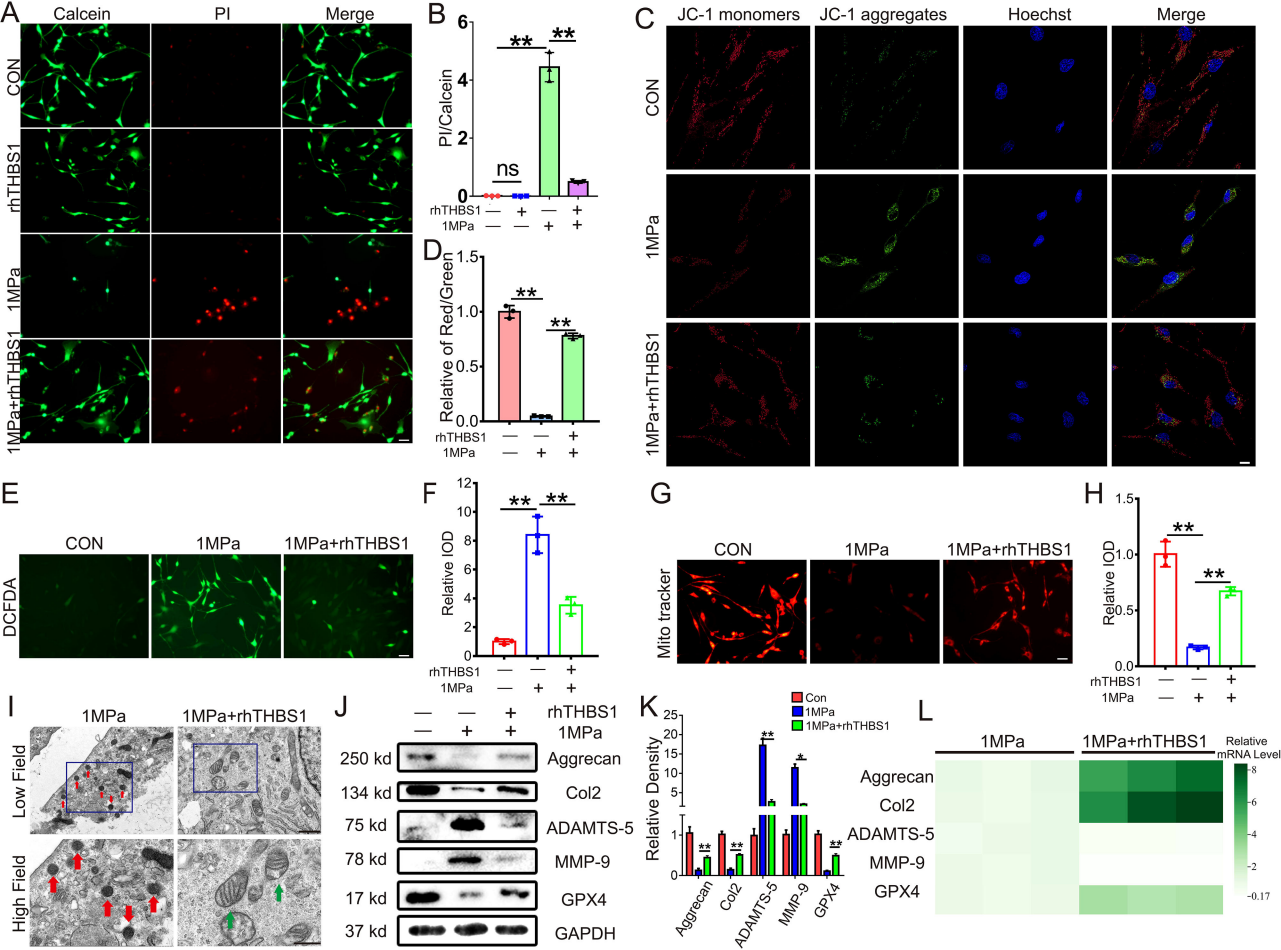
THBS1 inhibits chondrocytes ferroptosis induced by excessive mechanical stress. Human chondrocytes were stimulated at 1MPa mechanical stress for 2 hours with or without rhTHBS1 protein(100ng/ml). Relevant tests were performed 24 hours after the end of mechanical stress stimulation. **(A)** The cell death ratio of chondrocytes was tested by cell death/live analysis. Scale bar = 50 μm. **(B)** The cell number of PI (red fluorescence)/calcein (green fluorescence) reflected the cell death ratio (n=3 for each group). **(C)** Mitochondrial membrane potential was detected by JC-1 assay. Scale bar = 10 μm. **(D)** The relative IOD ratio of red fluorescence to green fluorescence was used for quantitative analysis (n=3 for each group). **(E)** Representative images of ROS levels in chondrocytes. Scale bar = 50 μm. **(F)** Quantitative analysis of fluorescence intensity (n=3 for each group). **(G)** Representative fluorescence images of mitochondria in chondrocytes. Scale bar = 50 μm. **(H)** Quantitative analysis of fluorescence intensity (n=3 for each group). **(I)** Representative TEM images of chondrocytes of indicated groups. (n=3 for each group). Green arrows show the normal mitochondria. Red arrows show the shrunken mitochondria. Scale bars, 1 μm (low field), 500 nm (high field). **(J)** Western blot (WB) analysis of Aggrecan, Col2, ADAMTS-5, MMP-9 and GPX4. **(K)** Quantification of WB analysis (n=3 for each group). **(L)** Real-time PCR of Aggrecan, Col2, ADAMTS-5, MMP-9 and GPX4 in chondrocytes of the indicated groups. (n=3 for each group). Data were presented as the mean ± SD. NS P>0.05 *P<0.05, **P<0.01.

To elucidate the specific mechanism by which THBS1 delays osteoarthritis and the reasons for its differential expression under mechanical stress, we constructed an *in vitro* mechanical stress stimulation model using mouse chondrocytes. Initially, we subjected mouse chondrocytes to an abnormal stress stimulus of 1 MPa. Under this abnormal stress, a significant number of chondrocytes underwent cell death. However, the addition of rhTHBS1 protein effectively rescued the chondrocytes from death induced by the abnormal mechanical stress (**Figures 3A, B**). JC-1 staining results indicated that abnormal mechanical stress impaired mitochondrial activity, but supplementation with rhTHBS1 restored this activity (**Figures 3C, D**). Furthermore, abnormal mechanical stress resulted in an increase in ROS and a decrease in mitochondrial membrane potential in chondrocytes. The administration of rhTHBS1 led to a reduction in ROS levels and an enhancement of mitochondrial membrane potential (**Figures 3E–H**). We further confirmed that high mechanical stress induces mitochondrial shrinkage and membrane thickening in chondrocytes, indicating that these cells undergo ferroptosis. However, rhTHBS1 was able to reverse this phenomenon (**Figure 3I**),. At the protein expression level, abnormal mechanical stress reduced GPX4 levels, while supplementation with rhTHBS1 increased GPX4 expression. Additionally, THBS1 upregulated the expression of chondrocyte synthesis markers Col2 and Aggrecan, while simultaneously suppressing the expression of degradation markers MMP-9 and ADAMTS-5 (**Figures 3J, K**). At the transcription level, abnormal mechanical stress inhibited the transcription levels of Col2, Aggrecan, and GPX4, while increasing the transcription levels of MMP-9 and ADAMTS-5. However, supplementation with rhTHBS1 restored the transcription levels of Col2, Aggrecan, and GPX4, and inhibited the transcription levels of MMP-9 and ADAMTS-5 (**Figure 3L**).

Additionally, we generated cartilage-specific GPX4 knockout mice (Col2a1-CreERT, GPX4^flox/flox^) to investigate the relationship between THBS1 and chondrocytes ferroptosis by intra-articular injection of rhTHBS1 protein. The breeding strategy and genotyping process are shown in **Supplementary Figure S1**. As illustrated in **Supplementary Figures S2B-D**, GPX4cKo mice displayed significantly reduced GPX4 expression and transcription levels in knee cartilage tissue following tamoxifen induction. Notably, our previous research indicated that THBS1 overexpression alleviated surgery-induced osteoarthritis; however, after GPX4 knockout in chondrocytes, THBS1 was unable to effectively reduce osteophyte formation (**Supplementary Figures S2E, F**), cartilage loss was exacerbated, and cartilage damage severity increased (**Supplementary Figures S2G, H**). These results indicate that the chondroprotective effect of THBS1 depends on GPX4-mediated chondrocytes ferroptosis.

We also validated the regulatory role of THBS1 in RSL3-induced ferroptosis in chondrocytes. Following RSL3 treatment, mitochondrial activity decreased significantly; however, supplementation with THBS1 effectively restored mitochondrial activity (**Supplementary Figures S3A, B**). Additionally, RSL3 reduced GPX4 expression within chondrocytes, while THBS1 supplementation restored GPX4 levels (**Supplementary Figures S3C, D**).

### The interaction between THBS1 and integrinαVβ1 is enhanced under mechanical stress

**Figure 4:**
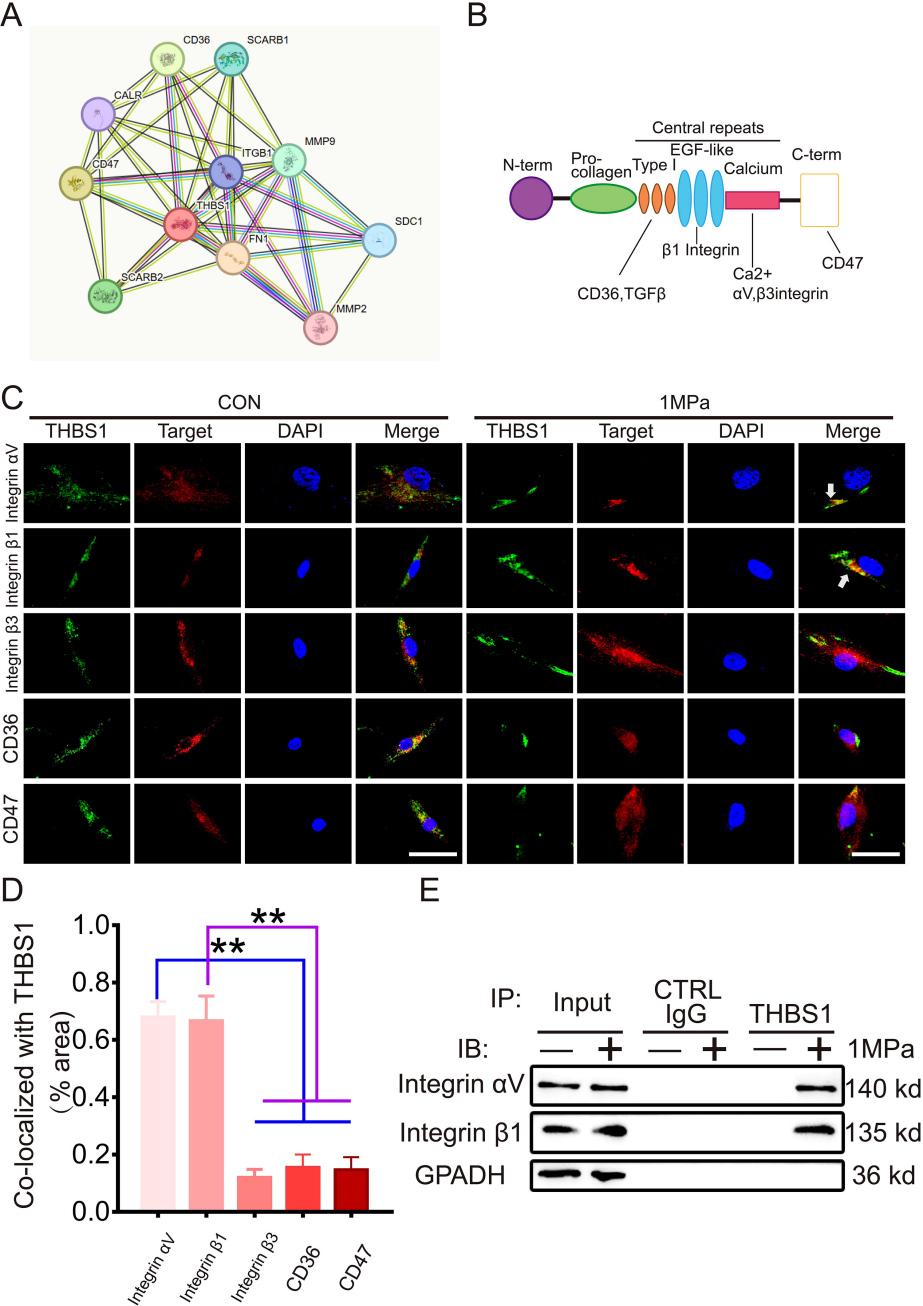
The interaction between THBS1 and IntegrinαVβ1 is enhanced under mechanical stress. **(A)** Interaction network of differential genes, each circle represented a gene, and the size of the circle represented the core level of the gene. The larger the circle, the higher the core level the gene had in the network. **(B)** Cartoon shows various binding domains of THBS1 and its receptors. **(C)** Representative immunostaining of THBS1 showing colocalization with integrins αv and β1 (white arrows) but not with integrin β3, CD47, or CD36 under mechanical stress (n = 6). Scale bars, 50 μm. **(D)** Quantification of colocalization of THBS1 with molecules shown in C using Image J software. **(E)** Immunoprecipitation (IP) with anti-THBS1 or control immunoglobulin G (IgG) followed by Western blotting for integrins αv and β1 (n = 3). IB, immunoblot. Data were presented as the mean ± SD. **P<0.01.

To elucidate the mechanism by which THBS1 regulates chondrocytes ferroptosis induced by mechanical stress, we analyzed the interaction network between THBS1 and various matrix interaction proteins using the STRING website (**Figure 4A**). THBS1 interacts with multiple proteins in this context, including integrinαV, integrin β1, integrin β3, CD36, and CD47. Given that THBS1 has well-characterized surface receptors (**Figure 4B**), we investigated which molecules interact with THBS1 during mechanical stress. We performed IF detection immediately after chondrocytes were subjected to mechanical stress stimulation *in vitro*. Immunofluorescence staining revealed that THBS1 co-localized with integrinαV and integrin β1, but not with integrin β3, CD36, and CD47 (**Figures 4C, D**). Furthermore, we conducted co-immunoprecipitation (CO-IP) experiments. The THBS1 antibody was utilized to precipitate cell lysates under both static and high mechanical stress conditions, followed by WB detection to confirm the direct binding of THBS1 to integrinαV and integrin β1 under high mechanical stress (**Figure 4E**).

### THBS1 inhibits mechanical stress-induced chondrocytes ferroptosis through integrinαVβ1

**Figure 5:**
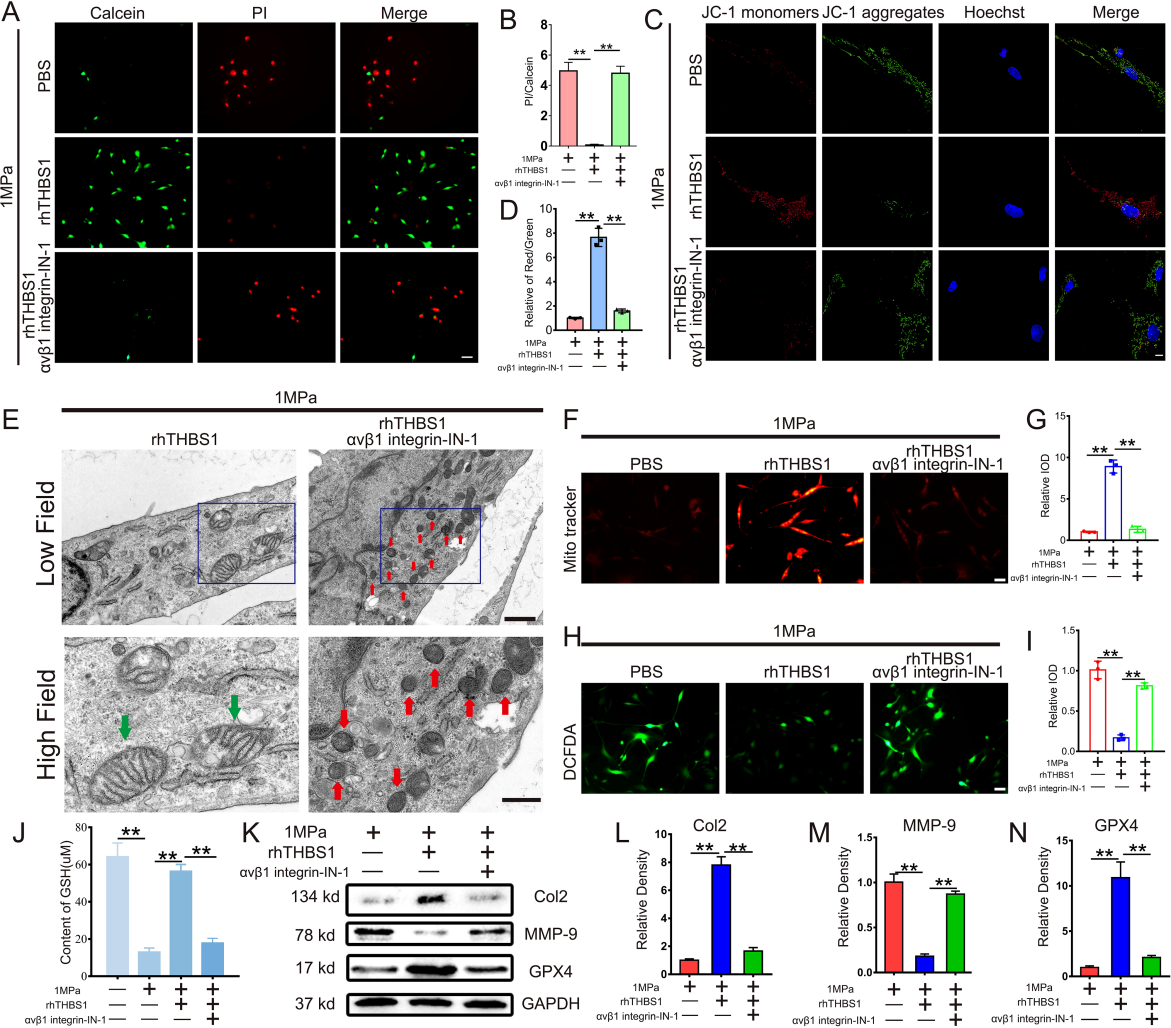
THBS1 inhibits mechanical stress-induced ferroptosis in chondrocytes through integrinαVβ1. Human chondrocytes were stimulated at 1MPa mechanical stress for 2 hours with or without integrinαVβ1 inhibitor αVβ1 integrin-IN- 1(100ng/ml) and rhTHBS1(100ng/ml). Relevant tests were performed 24 hours after the end of mechanical stress stimulation. **(A)** The cell death ratio of chondrocytes was tested by cell death/live analysis. Scale bar = 50 μm. **(B)** The cell number of PI (red fluorescence)/calcein (green fluorescence) reflected the cell death ratio (n=3 for each group). **(C)** Mitochondrial membrane potential was detected by JC-1 assay. Scale bar = 10 μm. **(D)** The relative IOD ratio of red fluorescence to green fluorescence was used for quantitative analysis (n=3 for each group). **(E)** Representative TEM images of chondrocytes of indicated groups. (n=3 for each group). Green arrows show the normal mitochondria. Red arrows show the shrunken mitochondria. Scale bars, 1 μm (low field), 500 nm (high field). **(F)** Representative fluorescence images of mitochondria in chondrocytes. Scale bar = 50 μm. **(G)** Quantitative analysis of fluorescence intensity (n=3 for each group). **(H)** Representative images of ROS levels in chondrocytes. Scale bar = 50 μm. **(I)** Quantitative analysis of fluorescence intensity (n=3 for each group). **(J)** The expression of GSH in chondrocytes of indicated groups was detected by ELISA (n=3 for each group). **(K)** Western blot (WB) analysis of Col2, MMP-9 and GPX4. **(L-N)** Quantification of WB analysis (n=3 for each group). Data were presented as the mean ± SD. **P<0.01.

To investigate whether THBS1 regulates mechanical stress-induced chondrocytes ferroptosis via integrinαVβ1, we administered the integrinαVβ1 inhibitor αVβ1 integrin-IN-1. Following the blockade of integrinαVβ1, THBS1 was unable to restore the diminished chondrocyte activity resulting from high mechanical stress (**Figures 5A, B**). Additionally, the protective effect of THBS1 on mitochondrial activity was also decreased upon the introduction of the integrinαVβ1 inhibitors (**Figures 5C, D**). Transmission electron microscopy analysis revealed that rhTHBS1 could not inhibit mechanical stress-induced chondrocytes ferroptosis after integrinαVβ1 was inhibited (**Figure 5E**). Under conditions of high mechanical stress, rhTHBS1 enhanced the mitochondrial membrane potential in chondrocytes and reduced intracellular ROS levels; however, this effect was obstructed by the application of integrinαVβ1 inhibitors (**Figures 5F-I**). Further analysis indicated that rhTHBS1 plays a crucial role in maintaining GSH levels in chondrocytes subjected to high mechanical stress, with a significant reduction in GSH levels observed following the addition of integrinαVβ1 inhibitors (**Figure 5J**). In terms of protein expression, under high mechanical stress, rhTHBS1 elevated the expression of Col2 and GPX4 while decreasing the expression of MMP-9. The introduction of integrinαVβ1 inhibitors resulted in reduced levels of Col2 and GPX4, alongside an increase in MMP-9 expression (**Figures 5K-N**).

### THBS1 inhibits chondrocyte ferroptosis through the integrinαVβ1/YAP pathway

**Figure 6:**
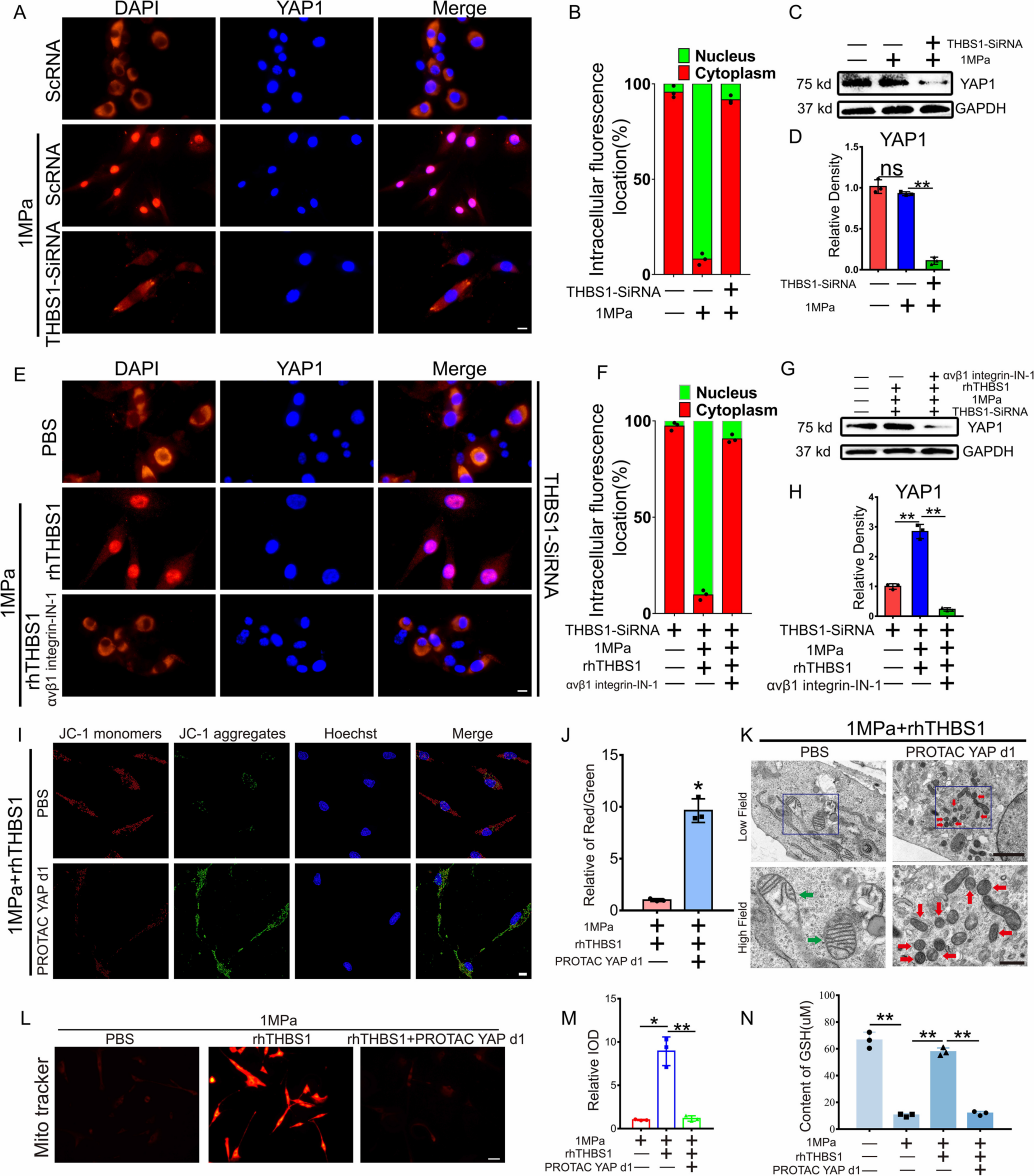
THBS1 inhibits chondrocyte ferroptosis through the integrinαVβ1/YAP pathway. THBS1 knockout chondrocytes and normal chondrocytes were subjected to 1 MPa mechanical stress for 2 hours, with or without the addition of YAP1 inhibitor PROTAC YAP d1 (20μM) and rhTHBS1(100ng/ml). Immediately following stimulation, IF detection was performed. Additional analyses were conducted after chondrocytes were further incubated with or without YAP1 inhibitor PROTAC YAP d1 (20μM) and rhTHBS1(100ng/ml) for 24 hours post-stimulation. **(A)** Representative image of chondrocytes undergoing IF detection immediately after mechanical stress stimulation. YAP (red) and DAPI (blue) are also shown. Scale bar = 20 μm. **(B)** Quantification of YAP localization. In each experiment 150 to 200 cells were evaluated (n = 3). YAP localization in the nucleus (green) or cytoplasm (red) is shown. **(C)** Western blot (WB) analysis of YAP1. **(D)** Quantification of WB analysis (n=3 for each group). **(E)** Representative image of chondrocytes undergoing IF detection immediately after mechanical stress stimulation. YAP (red) and DAPI (blue) are also shown. Scale bar = 20 μm. **(F)** Quantification of YAP localization. In each experiment 150 to 200 cells were evaluated (n = 3). **(G)** Western blot (WB) analysis of YAP1. **(H)** Quantification of WB analysis (n=3 for each group). **(I)** Mitochondrial membrane potential was detected by JC-1 assay. Scale bar = 10 μm. **(J)** The relative IOD ratio of red fluorescence to green fluorescence was used for quantitative analysis (n=3 for each group). **(K)** Representative TEM images of chondrocytes of indicated groups. (n=3 for each group). Green arrows show the normal mitochondria. Red arrows show the shrunken mitochondria. Scale bars, 5.0 μm (low field), 500 nm (high field). **(L)** Representative fluorescence images of mitochondria in chondrocytes. Scale bar = 50 μm. **(M)** Quantitative analysis of fluorescence intensity (n=3 for each group). **(N)** The expression of GSH in chondrocytes of indicated groups was detected by ELISA (n=3 for each group). Data were presented as the mean ± SD. NS P>0.05 *P<0.05, **P<0.01.

The activation of the integrin system induces subsequent changes in downstream molecules. In this study, we analyzed the activation of YAP1, a molecule closely associated with stress transduction. We inhibited the expression of THBS1 with SiRNA to observe the regulatory effect of THBS1 on YAP1 under mechanical stress. SiRNA can effectively inhibit THBS1 transcription level and expression (**Supplementary Figures S4A-C**). Immunofluorescence (IF) analysis demonstrated that mechanical stress promoted the nuclear translocation of YAP1, while the knockout of THBS1 diminished the extent of YAP1’s nuclear translocation (**Figures 6A, B**). WB detection revealed that the knockdown of THBS1 resulted in a reduced expression level of YAP1 under conditions of high mechanical stress (**Figures 6C, D**). Furthermore, the addition of rhTHBS1 and integrinαVβ1 inhibitors indicated that rhTHBS1 facilitated the nuclear translocation of YAP1, whereas the blockade of integrin signaling decreased the degree of YAP1 nuclear translocation (**Figures 6E, F**). Concurrently, rhTHBS1 elevated the expression level of YAP1, which was significantly reduced following the application of the integrinαVβ1 inhibitor (**Figures 6G, H**). The protective effect of rhTHBS1 on mitochondrial activity in chondrocytes subjected to high mechanical stress was found to be attenuated by YAP1 inhibitors (**Figures 6I, J**). Upon the introduction of the YAP1 inhibitor, rhTHBS1 was unable to prevent ferroptosis in chondrocytes induced by high mechanical stress (**Figure 6K**). Additionally, the mitochondrial membrane potential in chondrocytes was diminished following the administration of the YAP1 inhibitor (**Figures 6L, M**), and GSH levels in chondrocytes were significantly lowered after YAP1 inhibition (**Figure 6N**).

### THBS1-like peptide inhibited the progression of osteoarthritis and protected articular cartilage

**Figure 7:**
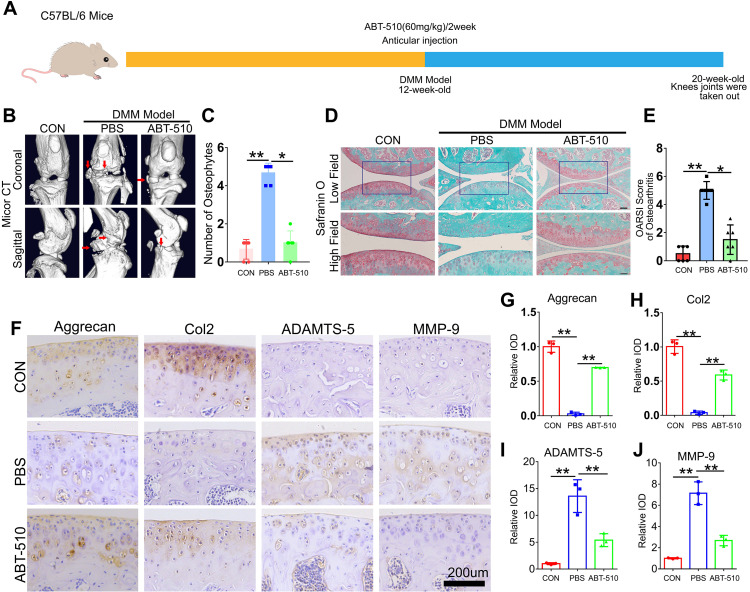
THBS1-like peptide has been shown to significantly inhibit the progression of osteoarthritis and protect articular cartilage. **(A)** Flowchart of animal experiment. **(B)** Representative images of Micro-CT of the indicated groups (n=6). Arrows show the formation of osteophytes. **(C)** Osteophyte number assay based on Micro-CT (n=6 for each group). **(D)** Representative images of safranin O staining of the indicated groups. Scale bars, 100 μm (low field), 50 μm (high field). **(E)** Osteoarthritis Research Society International (OARSI) score of OA based on the results of safranin O staining (n=6 for each group). **(F)** Immunohistochemical assay of Aggrecan, Col2, ADAMTS-5 and MMP-9 in articular cartilage of the indicated groups. Scale bars 200 μm. **(G-J)** Quantification of immunohistochemical analysis (n=3 for each group). Data were presented as the mean ± SD. *P<0.05, **P<0.01.

To evaluate the therapeutic potential of THBS1 for OA, we selected ABT-510, a synthetic THBS1-like peptide. At 12 weeks of age, a destabilization of the medial meniscus (DMM) model was established in the left knee joint of mice, followed by the injection of ABT-510. The control group received an equivalent volume of PBS. Injections were administered bi-weekly for a total of four consecutive doses. After an 8-week period, knee joint tissue from the mice was harvested (**Figure 7A**). Micro CT imaging revealed that ABT-510 significantly reduced osteophyte formation in the knee joint tissue (**Figures 7B, C**). SO, staining indicated that ABT-510 protected articular cartilage and lowered the OARSI scores (**Figures 7D, E**). Additionally, IHC staining analysis demonstrated that ABT-510 decreased catabolic activity and promoted anabolic processes in knee joint cartilage tissue (**Figures 7F-N**).

## Discussion

Mechanical signals play a crucial role as an initiating factor in various physiological and pathological processes in different types of cells (25–29). Decades of research have demonstrated that the role of mechanical stress in OA pathobiology is not merely mechanical “wear and tear”. Rather, mechanical stress influences the development of osteoarthritis by activating mechanosensitive cellular signaling pathways, which in turn disrupt the balance of anabolic and catabolic enzymes in chondrocytes (30–33).

Although osteoarthritis is a multifaceted disease involving both articular cartilage and synovium (34), the degeneration of articular cartilage remains a significant pathological change associated with OA (35). Chondrocytes are responsible for the secretion and turnover of ECM components, making them essential for maintaining ECM homeostasis. Numerous studies have demonstrated that disorders in the synthesis and metabolism of chondrocytes, as well as abnormal cellular activity, can lead to cartilage degeneration and contribute to the development of osteoarthritis (36).

Given the central regulatory role of chondrocytes in cartilage tissue and their function as key mediators in response to mechanical stress (37), our previous study identified that abnormal mechanical stress can induce ferroptosis in chondrocytes (4), thereby exacerbating the progression of OA. Ferroptosis, a recently discovered form of cell death, results in mitochondrial dysfunction and oxidative damage to lipid membranes (38). In this study, we further validated the differential changes between the loading and unloading regions of cartilage in knee OA patients, specifically noting that chondrocytes in the loading region exhibited characteristic features of ferroptosis. Additionally, we observed the differential expression of THBS1 in the loading regions. Although mechanical stress enhanced the transcriptional level of THBS1 in the *in vitro* chondrocyte compression model, this increase is likely a reactive response induced by mechanical stress stimulation. Previous studies have also confirmed that THBS1 exhibits a reactive elevation during the early stages of osteoarthritis (10). In a short, these findings suggests that THBS1 may be involved in chondrocyte damage induced by mechanical stress during OA progression.

THBS1 is an extracellular matrix protein that plays a critical role in cell-matrix interactions (5, 39), which are essential for cells to sense and respond to mechanical stress. THBS1 is also associated with the development of musculoskeletal system diseases. Multiple studies have shown that THBS1 may regulate bone regeneration (40, 41). Blocking TSP1 can alleviate osteoclast-mediated hypercalcemia in mice (42, 43). And several studies have also shown that THBS1 may be related to cartilage degeneration in osteoarthritis (10–12, 44). In this study, we explored the role of THBS1 in OA progression by transfecting adeno-associated virus to overexpress THBS1. Our findings indicated that overexpression of THBS1 effectively slowed the progression of knee OA in rat, promoting anabolic processes in chondrocytes while inhibiting catabolic activity. These data suggest that THBS1 may serve as a potential therapeutic target for OA.

Next, we investigated the specific mechanisms by which THBS1 protects joint cartilage and slows the progression of OA. In an *in vitro* chondrocyte compression model, THBS1 effectively protected chondrocytes from damage induced by excessive mechanical stress. Moreover, THBS1 significantly reduced intracellular ROS levels and enhanced mitochondrial activity in chondrocytes. Additionally, THBS1 upregulated the expression of GPX4, a key regulator of ferroptosis known for its protective role against oxidative damage (45), thereby preventing the occurrence of ferroptosis in chondrocytes.

THBS1 is a multi-modular protein containing several distinct domains that interact with various cell receptors to perform critical functions (46). In this study, we analyzed the potential binding proteins involved in THBS1-mediated cell-matrix interactions through a protein interaction network. We also examined the binding of several key proteins to THBS1. The results showed that under high mechanical stress, the interaction between THBS1 and Integrin αVβ1 was enhanced. When Integrin αVβ1 was blocked, the protective effect of THBS1 on inhibiting chondrocyte ferroptosis disappeared. Previous studies have also demonstrated that THBS1 is an important ligand for the Integrin system, interacting with various Integrin subtypes (such as αVβ1, α3β1, and α5β1) to play a crucial role in cell-cell and cell-matrix interactions (6, 7). The Integrin system, a family of transmembrane receptor proteins, mediates cell adhesion and signal transduction between cells and the extracellular matrix, including mechanical signal transduction, and is essential for biological processes such as cell migration, proliferation, and differentiation (47). Furthermore, studies have shown that mechanical stress is a key factor in regulating the binding of THBS1 to Integrins (9). Under mechanical stress, the THBS1 protein in the extracellular matrix undergoes stretching and twisting, leading to conformational changes that expose its Integrin-binding sites. This structural alteration increases the affinity of THBS1 for Integrins, thereby promoting their interaction.

The Integrin system acts as a bridge between the extracellular matrix and the cell interior, serving as a critical signaling pathway that regulates the activity of various intracellular signaling cascades (8). Studies have shown that the interaction between Integrins and the extracellular matrix can activate the FAK signaling pathway, which subsequently influences the nuclear translocation of YAP (9, 48–50). YAP, a transcription factor that plays a key role in biological processes such as cell growth, proliferation, and differentiation, is also regulated by mechanical stress (51–53). Our research demonstrated that under mechanical stress, THBS1 promotes YAP nuclear translocation, and supplementation with THBS1 further enhances YAP expression levels. However, when Integrin was blocked, YAP nuclear translocation was abolished. These data suggest that THBS1 inhibits mechanical stress-induced ferroptosis in chondrocytes via the Integrin/YAP pathway. GSH homeostasis is essential for the proper production and function of GPX4 (54). We found that THBS1, through its interaction with the Integrin/YAP pathway, helps maintain GSH levels, thereby sustaining GPX4 expression and function. This, in turn, facilitates the clearance of excess lipid peroxides within chondrocytes, preventing ferroptosis in these cells.

**Figure 8:**
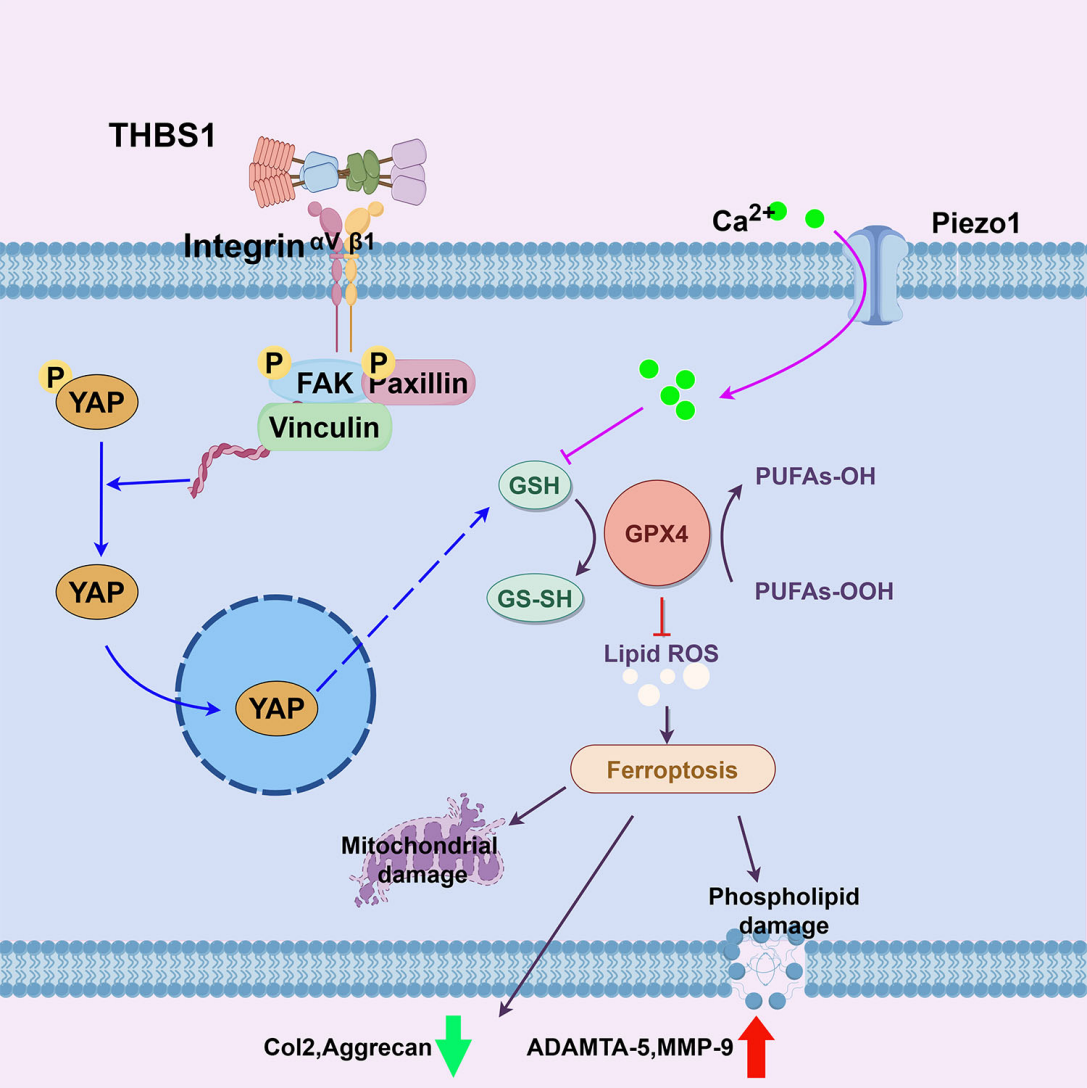
Schematic diagram of this study.

Based on the current research and previous studies, we propose a novel therapeutic target for osteoarthritis. In the presence of abnormal mechanical stress, supplementation with THBS1 or similar peptides (e.g., ABT-510) could activate the Integrin/YAP system, preventing the stress-induced reduction in GSH levels, maintaining GPX4 activity and expression, and thereby preventing ferroptosis in chondrocytes (**Figure 8**). This approach would help sustain anabolic processes in chondrocytes while inhibiting catabolic activity.

## Conclusion

In conclusion, this study explored therapeutic targets for ferroptosis in chondrocytes induced by abnormal mechanical stress. We provided preliminary evidence that THBS1 plays a protective role in preventing chondrocyte ferroptosis and slowing the progression of OA. Additionally, we investigated the specific mechanisms by which THBS1 inhibits ferroptosis in chondrocytes. These findings may offer insights into potential therapeutic interventions for OA and other diseases influenced by mechanical stress.

## Data Availability

All data presented in the study are included in the article/**Supplementary Material**. All the data have been uploaded.
