# Supplementary material for: Thrombospondin-1 mitigates osteoarthritis progression by inhibiting mechanical stress-induced chondrocyte ferroptosis via the integrin/YAP pathway
Source: Front Immunol. 2025 May 22;16:1577234. doi: 10.3389/fimmu.2025.1577234 (PMC12137073; doi:10.3389/fimmu.2025.1577234)
Supplement: Supplementary file 1 [file DataSheet1.docx]

**Thrombospondin-1 Mitigates Osteoarthritis Progression by Inhibiting Mechanical stress-induced Chondrocyte Ferroptosis via the Integrin/YAP Pathway**

Shaoyi Wang^1,2^, Xiaocong Zhou^3^, Fujian Zhang^1^, Haoxin Zhai^1^, Yuanqiang Zhang^1,2, #^, Yongyuan Guo^1, #^.

Affiliations:

1. Department of Orthopaedic Surgery, Qilu Hospital, Cheeloo College of Medicine, Shandong University, Jinan, Shandong, 250012, P. R. China.

2. Qilu Hospital of Shandong University Spine and Spinal Cord Disease Research Center- ICMRS Collaborating Center for Orthopaedic translational Research, Shandong University, Jinan, Shandong, 250012, P.R. China.

3. Health Management Centre, The First Affiliated Hospital of Shandong First Medical University, Jinan, Shandong, 250012, P. R. China.

#To whom correspondence should be addressed: Yuanqiang Zhang, Department of Orthopaedic Surgery, Qilu Hospital, Cheeloo College of Medicine, Shandong University, 107 Wenhuaxi Road, Jinan, P. R. China 250012. Email: drzhyq@126.com

Yongyuan Guo, Department of Orthopaedic Surgery, Qilu Hospital, Cheeloo College of Medicine, Shandong University, 107 Wenhuaxi Road, Jinan, P. R. China 250012. Email: 201362006478@email.sdu.edu.cn


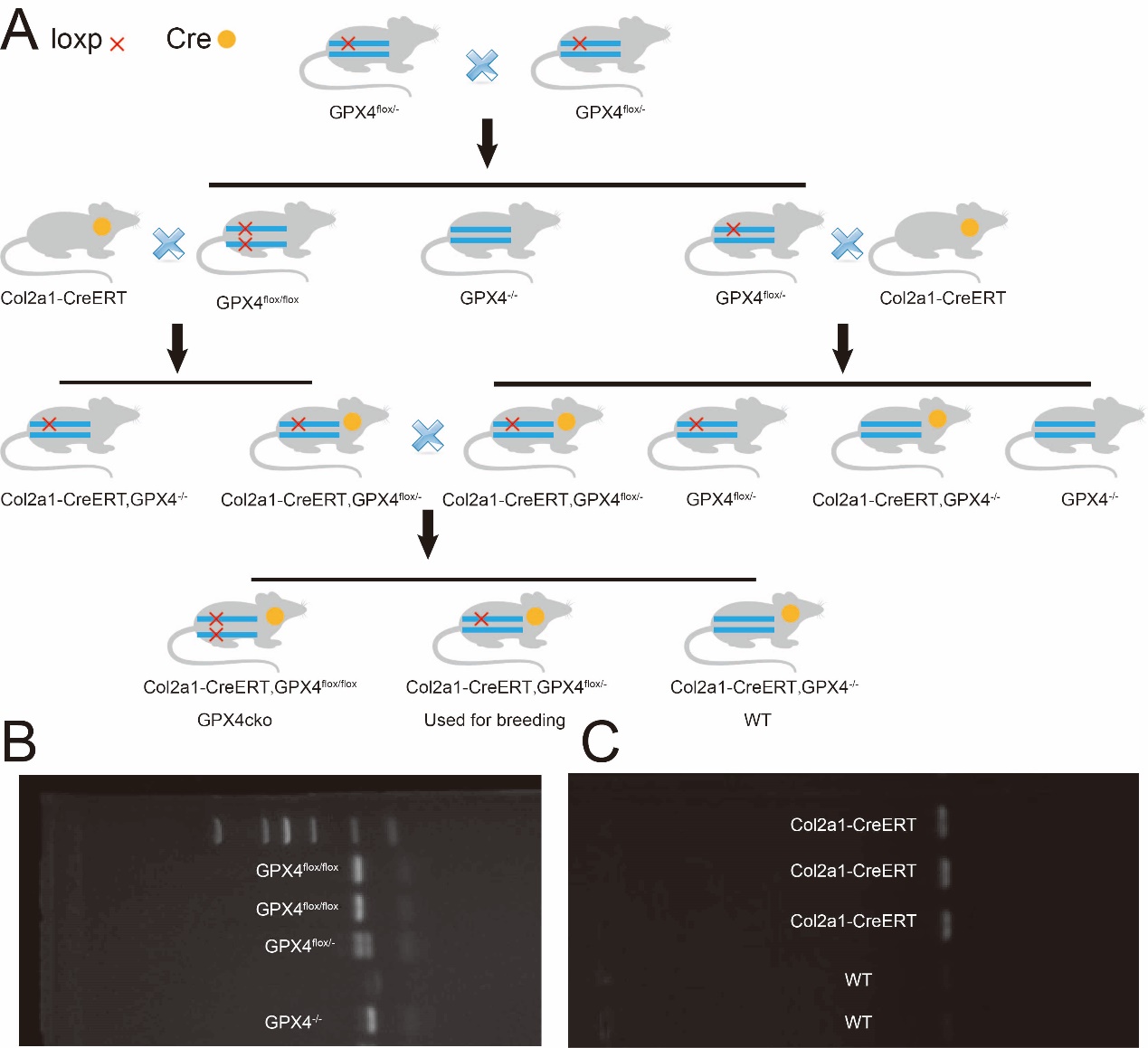


Figure S1. Mice breeding strategy and fund identification results.

(A). Mouse breeding strategies. (B). Gene identification results: Homozygotes (GPX4^flox/flox^) :238bp. Heterozygotes (GPX4^flox/+^) 238/204bp. Wild type (GPX4^+/+^) 204bp. Part of gene identification results. (C). Gene identification results: Col2a1-CreERT: 358bp. Wild type: No stripe.


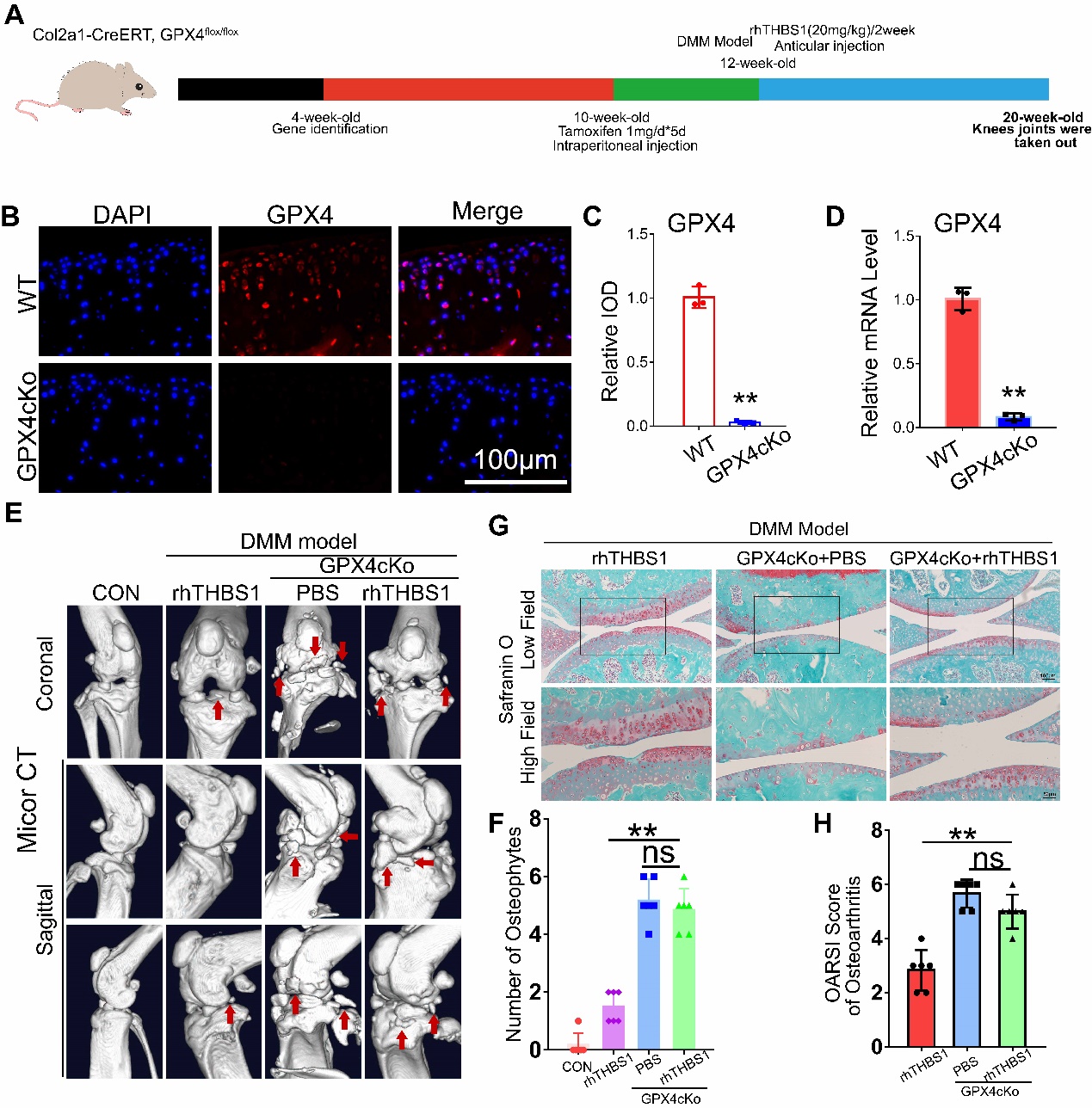


Figure S2. The protective effect of THBS1 on the cartilage of GPX4 knockout mice was decreased.

(A). Flowchart of animal experiment. (B). Representative immunofluorescence images of GPX4 in articular cartilage of the indicated groups. Scale bars, 100 μm. (C). Quantification of immunofluorescence analysis (n=3 for each group). (D). Real-time PCR of GPX4 in articular cartilage. (E). Representative images of Micro-CT of the indicated groups (n=6). Arrows show the formation of osteophytes. (F). Osteophyte number assay based on Micro-CT (n=6 for each group). (G). Representative images of safranin O staining of the indicated groups. Scale bars, 100 μm (low field), 50 μm (high field). (H). Osteoarthritis Research Society International (OARSI) score of OA based on the results of safranin O staining (n=6 for each group). Data were presented as the mean ± SD. *P<0.05, **P<0.01.


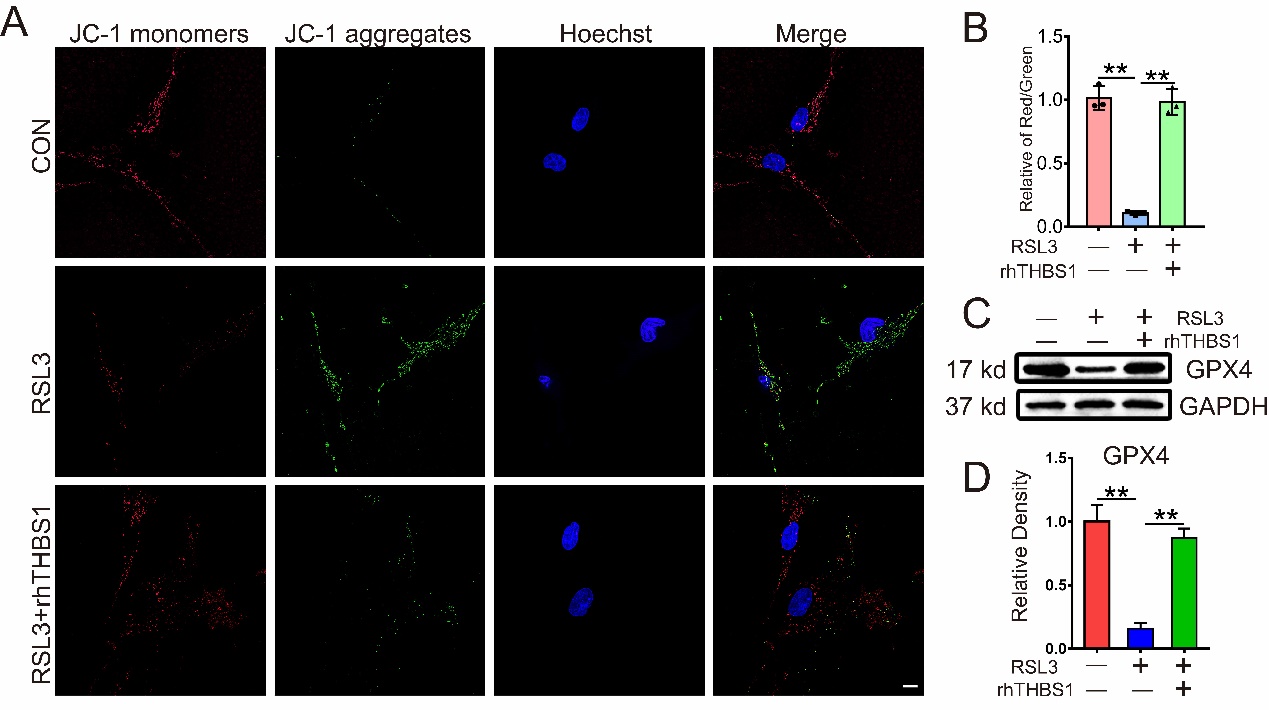


Figure S3. THBS1 inhibited RSL3-induced ferroptosis chondrocyte.

Chondrocytes were incubated with RSL3 (10μM) and with or without rhTHBS1 ((100ng/ml) 24 hours, then test were performed. (A). Mitochondrial membrane potential was detected by JC-1 assay. Scale bar = 10 μm. (B). The relative IOD ratio of red fluorescence to green fluorescence was used for quantitative analysis (n=3 for each group). (C). Western blot (WB) analysis of GPX4. (D). Quantification of WB analysis (n=3 for each group). Data were presented as the mean ± SD. **P<0.01.


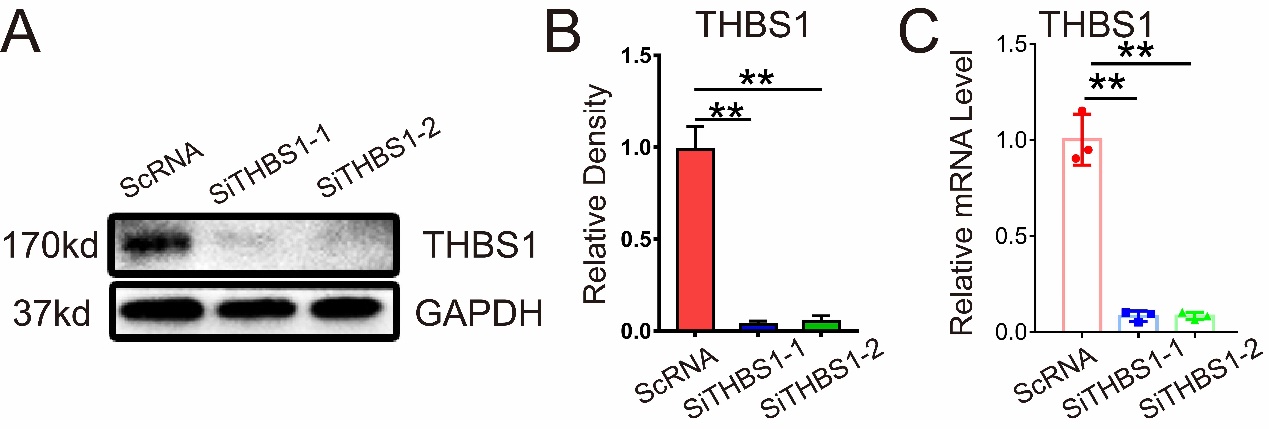


Figure S4. Knockout efficiency of THBS1

(A). Western blot (WB) analysis of THBS1. (B). Quantification of WB analysis (n=3 for each group). (C). Real-time PCR of GPX4 in chondrocytes. Data were presented as the mean ± SD. **P<0.01.

**Table S1: Gene identification primers**

| Target | Forward Primers,5’-3’ | Reverse Primers,5’-3’ |
| --- | --- | --- |
| GPX4flox | TCCATTGGTCGGCTGCGTGAGG | ACCCTGGATACGGTGACCCGAC |
| Col2a1-CreERT | CACTGCGGGCTCTACTTCAT | ACCAGCAGCACTTTTGGAAG |

**Table S2 Murine OARSI score system**

| Grade | Osteoarthritic damage |
| --- | --- |
| 0 | Normal cartilage without damage |
| 0.5 | Loss of Safranin-O staining while no detectable structural change |
| 1 | Small fibrillation |
| 2 | Vertical damage of cartilage limited to superficial layer |
| 3 | Vertical damage, no more than 25% of the cartilage surface |
| 4 | Vertical damage, 25-50% of the cartilage surface |
| 5 | Vertical damage, 50-75% of the cartilage surface |
| 6 | Vertical damage, more than 75% of the cartilage surface |
